# Supplementary material for: Incidence, risk factors, and feto-maternal outcomes of inappropriate birth weight for gestational age among singleton live births in Qatar: A population-based study
Source: PLoS One. 2021 Oct 28;16(10):e0258967. doi: 10.1371/journal.pone.0258967 (PMC8553085; doi:10.1371/journal.pone.0258967)
Supplement: Attachment — Submitted filename: Response.docx [file pone.0258967.s006.docx]

**Point by point response**

PONE-D-21-07926

Incidence, Risk Factors, and Feto-Maternal Outcomes of Inappropriate Birth Weight for Gestational Age: A Population-Based Study

PLOS ONE

We would like to take this opportunity to thank the editor and the reviewers for their comments and for taking the time to evaluate our manuscript. We have revised the article accordingly and point-by-point responses to the comments are reported below. All the changes made to the manuscript are highlighted in the Main Document using “Track Changes” tool.

Please note that other reviewers have requested other changes, and thus these will also appear in the manuscript.

Please follow the clean version of the manuscript with track changes turned “Off” for the indicated line numbers.

Reviewer #1: General comments

- The study would be of greater contribution if provides the performance of risk assessment models based on the significant risk factor. Risk-stratified algorithms are of great interest in clinical practice. Additionally, consider providing population prevalence and population attributable fractions for the associated factors.

Response: We have done all of these extra analyses including risk stratified algorithm using Kaplan Meier analysis (please see statistical analysis subsection Page 11, lines 176-180, results section Page 29, lines 327-335). Population prevalence and population attributable fractions were also calculated and added to Table 2, Table S2, and Table S3 (please see statistical analysis subsection Page 11, lines 167-175, and results section Pages 16-19, and page 20, lines 253-260).

- Detailed description of the method for estimating gestational age is crucial. It is strongly recommended to report (Suppl Info) the proportion of women whose gestational age was estimated by each method – for instance, by 1) LMP only; 2) early US only; 3) LMP and early US; 4) LMP and late US; or 5) late US only.

Response: We agree about the need for detailed description of the method for estimating gestational age. Unfortunately, the Pearl-Peristat Study is an observational study primarily designed for broader maternal and neonatal outcomes in the perinatal period using routinely collected hospital data. Thus, the data was not exhaustive and we do not have access to such granular data which could have added value to our findings. We have added this issue also as a study limitation.

- Provide absolutes risks for the outcomes.

Response: we thank the reviewer for this suggestion, we calculated the absolute risks for SGA and LGA (presented in Table S2 and Table S3), and for Apgar score, NICU/death in LR/OT, and in-hospital mortality (Table S4-A and Table S4-B).

Title

- Make clear what is the setting of the study; which population is this study was related to?

Response: We changed the title to clarify the study setting and the study population.

Abstract

- (Background) Preferably, use inadequate than aberrant.

Response: We changed the word aberrant to abnormal. As the study is on both small-for-gestational age (SGA) and large-for-gestational age born infants, and thus we thought that the word abnormal might reflect the main variables (Page 3, line 2).

- What do you mean by “long-term complication during pregnancy”?

Response: We rephrased the sentence and removed long term complications during pregnancy (Page 3, lines 2,3).

- Describe the reference chart applied for classifying adequacy of birth weight.

Response: We added an explanation about the tool and methodology used to estimate the centiles and the adequacy of birth weight (Pages 7,8 lines 90-l03).

- The sentence “Preterm birth significantly predicted SGA and LGA” did not seem accurate. Prediction implies diagnostic performance assessment, which was not the case in this analysis. Please, revise. Same for GDM and PGDM significantly predicted LGA.

Response: We changed the words “predicted” / “predict” to “associated with” across the manuscript.

- Avoid using abbreviations in the abstract, especially when they were not previously detailed (e.g., PGDM and LR/OT).

Response: We replaced the indicated abbreviations with the full terms (Page 3, line 15-19).

- How could SGA and LGA lead to c-section if they are defined by a postnatal condition (birth weight)?

Response: We have removed the regression analysis of delivery mode (vaginal vs. cesarean), and removed it from the table of outcomes (Table 3). We have only left the descriptive statistics in Table 1.

- Conclusion: The fact the this was the first study in Qatar does not necessarily novel contributions to the research field. In addition, it is not a reasonable conclusion foe the abstract.

Response: The indicated sentence has been removed from the abstract (Page 3).

Methods

- Consider using SGA- and LGA-related outcomes. Precious studies have shown that SGA and LGA may impact differently on perinatal outcomes (e.g. Neonatal Morbidity of Small- and Large-for-Gestational-Age Neonates Born at Term in Uncomplicated Pregnancies. Obstet Gynecol. 2017;130(3):511-9).

Response: We considered Apgar score, in-hospital mortality, admission to neonatal intensive care unit or death in labor room or operation theatre as outcomes, unfortunately we don’t have any other perinatal outcomes in the current paper. In future investigations, we will build up on the current analysis, and include more feto-maternal outcomes.

- Describe in more detail the tool used to estimate birth weight centiles. Several studies conducted in different populations concluded that chart-specific thresholds for a specific population should be considered in clinical practice, once different charts have different performance on identifying SGA and LGA babies. Why choosing this specific tool (Ref 34)?

Response: This tool provides a simple spreadsheet-based estimated fetal weight percentile calculator and corresponding R software package to encompass 6 fetal growth standards: the INTERGROWTH-21st, World Health Organization (WHO), National Institute of Child Health and Human Development (NICHD), Perinatology Research Branch (PRB/NICHD), and the Hadlock et al3 and Fetal Medicine Foundation (FMF) standards. These calculations take into account mother characteristics including ethnic/race group as well as height and weight. Our study included different ethnicities and nationalities. Thus, the tool we used takes into account the population (ethnic/race) that is investigated in the study.

We have added this information on how we estimate birth weight centiles in the methodology (please see Pages 7,8, lines 90-103.

- Definition of some variables are not clear enough. Please, make clear the definition for smoking (Have cessation during pregnancy been considered?), preterm history (any PTB or only spontaneous?), GDM (IADPSG criteria? ADA criteria? Local criteria?), maternal BMI (Self-reported weight? Have you also considered early pregnancy weight?).

Response: We have put more details about some of the indicated variables including GDM Page 9, lines 124-134), maternal BMI (Page 9, lines 135-140). and preterm history (Page 10, lines 141,142). Unfortunately, we don’t have data for smoking cessation during pregnancy.

- The risks for adverse outcomes according to gestational age may be different for the early, full and late terms. When calculating the risk ratios, consider using the three categories instead of only newborns delivered between 39 and 40 weeks.

Response: We calculated the relative risk ratios for “preterm” using “term” as a reference group, which included all deliveries at at 37 weeks’ and above (Page 8, lines 105-107).

We have done extra analysis and further categorized gestational age into: extreme-to-very preterm, moderate preterm, late preterm, and early term, vs. full term (Page 8, lines 105-109). Please see Tables S3 and S5, for the full univariate and multivariate analyses repeated with the gestational age groups categorized as indicated. The findings were incorporated into the results section, page 21, lines 262-272, and page 28, lines 304-322.

The percentiles calculated using the FETAL-GPSXL calculator, are up to 40 weeks only (280 days) (Page 7, line 92), it was not applicable to calculate the percentiles for “late term” from 41 weeks, 0 days' to 41 weeks, 6 days' gestation, and “post-term” at 42 weeks'.

- Birth weight categorized as low and normal seem useless.

Response: we agree with the reviewer, we deleted it from the neonatal factors.

Results

- Why using Odds ration instead of relative risk. Preferably, use aRR.

Response: We converted all ORs to RRs and presented them in Table 2, Table 3, and the supplementary materials (Table S2, Table S3, Table S4, and Table S5). We rephrased the abstract and results accordingly.

- Provide absolutes risks for the outcomes.

Response: we thank the reviewer for this suggestion, we calculated the absolute risks for SGA and LGA (presented in Table S2 and Table S3), and for Apgar score, NICU/death in LR/OT, and in-hospital mortality (Table S4-A and Table S4-B).

Reviewer #2: Thanks for the opportunity to review this manuscript where the aim was to assess the incidence, risk factors, and feto-maternal outcomes associated with small-forgestational age (SGA) and large-for-gestational age (LGA) infants.

Response: we would like to take this opportunity to thank the editor and the reviewers for their comments and for taking the time to evaluate our manuscript. We have revised the article accordingly and point-by-point responses to the comments are reported below. All the changes made to the manuscript are highlighted in the Main Document using “Track Changes” tool.

Please note that other reviewers have requested other changes and thus these will also appear in the manuscript.

Please follow the clean version of the manuscript with track changes turned “off” for the indicated line numbers.

Overall: It is well known both that SGA, LGA and gestational week will be associated to neonatal outcome. Even if this is the largest study made in Qatar, there are other studies with the same aim. Please clarify the novelty of this study and the reason it adds new knowledge.

Response: We have indicated in detail the novelty of our study in the introduction section (please see Page 6, lines 57-72) and discussion (please see Pages 32,33, lines 386-416).

Abstract:

1. For example, in this sentence and throughout the manuscript please use the word associations instead of predicted/predictors. Because what you have tested is the potential association, you havn’t performed any predictive models. A variable will not necessarily be a good predictor just because there is a significant association “GDM (aOR 1.45, 95% CI:1.13–1.86) and PGDM (aOR 3.51, 95% CI:2.08–5.92) significantly predicted LGA.”

Response: We changed the words “predicted” / “predict” to “associated with” across all the manuscript.

2. “Both SGA (aOR, 1.56; 95% CI:1.06–2.31) and LGA (aOR, 1.34, 95% CI:1.04–1.73) significantly lead to caesarean deliveries”, According to the previous reasoning, SGA and LGA was associated to cesarean deliveries, it is hard from observational studies to be able to demonstrate causal relationships.

Response: We agree with the reviewer, we rephrased this section (Page 3, lines 11-20). The abstract was rephrased due to the changes made in the statistical analysis according to the recommendations indicated by the reviewer.

Background:

3. Gestational age is a strong indicator (??) of birthweight and fetal growth, both of which are influenced by a combination of environmental and genetic influences.

Response: We rephrased this section following the reviewer’s comment, and following the description in the Canadian Perinatal Health Report, 2000, 2003 (1, 2) (Page 5, lines 44-46).

Material and methods:

4. The study is based on 14000 deliveries from one large hospital, please give us some more information about the population to make it easier to evaluate the external validity of the study to other populations.

Response: We have added more information about the population and the hospitals involved in the study (Page 7, lines 80-88).

5. What was gestational age based on? Ultrasound in gw 10-12? Ultrasound in gw 18-20? Period data?

Response: We have added the information about gestational age in the methodology section (please see Page 8, lines 104,105).

6. Throughout the world we use different scales fetal growth, hence SGA is not always the same in different countries. What is you definition of AGA/SGA/LGA based on? Which algorithm/model?

Response: We used the FETALGPSXL tool. This tool provides a simple spreadsheet-based estimated fetal weight percentile calculator and corresponding R software package to encompass 6 fetal growth standards: the INTERGROWTH-21st, World Health Organization (WHO), National Institute of Child Health and Human Development (NICHD), Perinatology Research Branch (PRB/NICHD), and the Hadlock et al3 and Fetal Medicine Foundation (FMF) standards. These calculations take into account mother characteristics including ethnic/race group as well as height and weight. Our study included different ethnicities and nationalities. Thus, the tool we used takes into account the population (ethnic/race) that is investigated in the study.

We have added this information on how we estimate birth weight centiles in the methodology (please see Pages 7,8, lines, 90-103).

7. What was the definition of GDM? Was OGTT done? Which glucose values were defined as GDM?

Response: We have added this information in the methodology section (please see Page 9, lines 124-134)

8. You write that you adjust the models for all significant predictors. There are potential confounders and potential mediators, however you have not done predicting models and therefor it is not possible to call the variables predictors. Some of the variables you adjust for, as gestational age and fetal sex for example will not be potential confounder, they will be potential mediators, meaning that a part of the effect on SGA/LGA will pass through that mediator. The mediator could potentially explain a part of the association.

Response: We thank the reviewer for his/her pertinent comment and completely agree with his/her point. We changed the words “predicted” / “predict” to “associated with” across the manuscript.

We have also rephrased the results section to indicate that GA and gender are mediators while the other variables are confounders associated to SGA/LGA (please see results section).

9. Please make the exposures in the regression clear, it is a bit hard to follow the reasoning.

Response: We have explained the regression analysis in the statistical analysis subsection in detail (please see Pages 10,11, lines 151-166). We have also rephrased the results section to further clarify the exposures (please see the results section). We added information to indicate in detail the adjusted factors (please see the footnotes of Table 2, Table 3, and also in the supplementary materials: Table S2, Table S3, Table S4, and Table S5).

Results:

10. Please change in the results so that confounders/mediators and so on is coherent.

Response: We have made the explanation in the results section clearer and coherent with regards to confounders and mediators (Page 15, 20, 21, and 28). We have also indicated this in the statistical analysis subsection (Page 10).

Discussion

11. You write “we took this a step further and combined gestational age with weight”. Please explain in what way you did this.

Response: We agree that this sentence is not clear, and thus we have removed it. We have now explained in detail the tool and methodology on how we calculated the fetal growth according to gestational age (please see (Pages 7,8 lines 90-l03).

Conclusion

12. In the conclusion you write: “The findings of this study should be applied in antenatal care; in particular nutritional surveillance, support, and monitoring should be controlled to reduce the burden of inappropriate fetal growth.” The results of your study are associations to SGA/LGA neonatals. That there are associations does not mean that we know the cause behind the associations or that we know what to do to decrease risks. Please keep the conclusion to what you have studied.

Response: The conclusion section was rephrased as indicated (Pages 33,34; lines 426-432).

**References**

1.	Health Canada. Canadian Perinatal Health Report, 2000. Ottawa, ON: Minister of Public Works and Government Services Canada, 2000.

2.	Health Canada. Canadian Perinatal Health Report, 2003. Ottawa, ON: Minister of Public Works and Government Services Canada, 2003.
