# Supplementary material for: Incidence, risk factors, and feto-maternal outcomes of inappropriate birth weight for gestational age among singleton live births in Qatar: A population-based study
Source: PLoS One. 2021 Oct 28;16(10):e0258967. doi: 10.1371/journal.pone.0258967 (PMC8553085; doi:10.1371/journal.pone.0258967)
Supplement: Attachment — Submitted filename: Point by Point Response.docx [file pone.0258967.s007.docx]

**Point by point response**

PONE-D-21-07926R1

Incidence, Risk Factors, and Feto-Maternal Outcomes of Inappropriate Birth Weight for Gestational Age Among Singleton Live Births in Qatar: A Population-Based Study

PLOS ONE

We would like to take this opportunity to thank the editor and the reviewers for their comments and for taking the time to evaluate our manuscript. We have revised the article accordingly and point-by-point responses to the comments are reported below. All the changes made to the manuscript are highlighted in the Main Document using “Track Changes” tool.

Please note that other reviewers have requested other changes, and thus these will also appear in the manuscript.

Please follow the clean version of the manuscript with track changes turned “Off” for the indicated line numbers.

Journal Requirements:

Please review your reference list to ensure that it is complete and correct. If you have cited papers that have been retracted, please include the rationale for doing so in the manuscript text, or remove these references and replace them with relevant current references. Any changes to the reference list should be mentioned in the rebuttal letter that accompanies your revised manuscript. If you need to cite a retracted article, indicate the article’s retracted status in the References list and also include a citation and full reference for the retraction notice.

Response: All references have been checked, all are complete and correct.

Additional Editor Comments (if provided):

The authors have addressed almost all comments, but some clarification is still needed.

Does this method consider the sex of the newborn? If so, please include it on page 7 lines 91-92.

Response: Yes, the method considers newborn sex, the suggested change has been made (page 8, line 105).

Page 8 line 108 the word preterm is missing “extreme to very... preterm”

Response: The suggested change has been made, the word “preterm has been added (page 8, line 118).

Regarding mother age classification, the term “normal age” is not quite accurate. So, I suggest the use of young adults, adolescents, and advanced maternal age.

Response: The suggested changes have been made, the terms have been changed in the text (page 9, line 126; page 15, line 211, page 15, line 220, page 21, line 258, main tables (Tables 1-3), and supplementary materials (Tables S1-S5).

Page 10, Lines 148 and 149 have the same meaning, please keep only one version of it.

148 “All categorical and binary variables were expressed as numbers and percentages”

149 “Variables were summarized using numbers and percentages”

Response: The suggested changes have been made (page 10, line 158).

The definition of mediator factor is a variable that is in the middle of the causal path of exposure and outcome. SGA birth is not a cause of sex, for example. So, please check the classification of preterm birth and sex as mediators in the models.

Response: We rephrased the section to clarify the exact analyses performed, indicating the exposure and outcomes clearly, with the mediators and confounders in each analysis (page 10; lines 161,162, page 11, lines 166, 167, 171). Also, we fully reviewed the results section and made the necessary changes regarding the use of the word “mediator” or “confounder”.

Please check table 3 adjusted value of SGA for In-hospital mortality, the RR change from 7.95 (4.7– 13.46) to 226.56 (3.47– 318.22). Is it right? Which variables are causing this change?

Response: The indicated adjusted RR (226.56 (3.47– 318.22), is after adjusting for the risk variables associated with SGA that were significant in the univariate analysis, with p-values <0.05, presented in Table 2, which are as follows: gestational age, maternal age, parity, nationality, education, diabetes status, chronic hypertension, early- or pre-pregnancy BMI, baby gender, and any chromosomal or congenital abnormalities (Table 3–A). The number of SGA who died in the hospital is very small (n: 22/882 (2.5%), and thus after adjusting for other risk variables, the RR became overestimated. We have indicated this as one of the study limitations in the discussion (Page 36, Line 465-467)

Reviewers' comments:

Reviewer #1:

- It is not clear which standardized birth weight chart was used to classify neonates into SGA, AGA and LGA. Why using six methods (charts) based on fetal growth/weight standards for classifying appropriateness of birth weight?

Response: We have used a tool encompassing 6 fetal growth standards, among which we chose the INTERGROWTH-21^st^ birth weight standard to calculate the percentiles. We have added a section in the introduction to explain in detail why we based our calculations on this particular reference (Pages 6,7; Lines 66-77). Also, we clarified it in the methods (Page 8, Lines 108-113).

- Surprisingly, the method used for classifying adequacy of birth weight resulted in 6% of SGA and 16% of LGA neonates. Considering that the expected rate would be 10% for SGA and LGA, why did you find such discrepancy? What are the implications for 1) the interpretation of your findings and 2) the generalizability of your study?

Response: We have added a section to discuss the comments indicated regarding the observed disparities, implications, interpretation, and generalizability (Pages 31,32, Lines 363-395).

- What contribution to clinical practice does your study give?

Response: We have added a section to further highlight the contribution of the study findings to clinical practice (page 36, lines 473-485).

Reviewer #2: Thanks for the opportunity to re-review this manuscript where the aim was to assess the incidence, risk factors, and feto-maternal outcomes associated with small-forgestational age (SGA) and large-for-gestational age (LGA) infants.

The comments have been adressed, however it is still confusing and un-clear with the exposure and outcome in the different analyses. You write:“Firstly, logistic regression analysis was performed for risk factors/confounders (demographic and medical factors) and mediators (prematurity and gender) of appropriateness of fetal growth for the GA groups (SGA/LGA vs. AGA).”

As I understand it you have used SGA/LGA as outcome in these analysis and different risk factors as exposure. Which variables are confounders/mediators will depend on which variable is exposure and which one is the outcome. If SGA/LGA is the outcome preterm birth will be a mediator. However, in the analyses of outcome: “Secondly, logistic regression was performed to investigate the outcomes of SGA and LGA including Apgar score, NICU/death in LR/OT, and in-hospital mortality. Multiple logistic regression was performed (including all significant confounders and mediators from the univariate analysis) to investigate the association of SGA/LGA with Apgar score, NICU/death in LR/OT, and in-hospital mortality as outcomes.” I guess that in these analyses SGA/LGA were the exposure and then preterm birth has another role, in this setting preterm birth will be a confounder (or in some cases a mediator if we believe that the SGA/LGA caused the premature birth) Gender will be a mediator in the first analyses and a confounder in the second analyses. Please, make it clear what the exposure and the outcome is in the different analyses.

Response: We agree and thus we rephrased the section to clarify the exact analyses performed, indicating the exposure and outcomes clearly, with the mediators and confounders in each analysis. We agree that both gender and preterm birth are confounders in the second analysis and not mediators, thus we deleted the word “mediators” from the second analysis (pages 10, 11; lines 161, 173), also, we fully reviewed the results section and made the necessary changes regarding the use of the word “mediator” or “confounder”.

I note that attributable fractions are added. For example you write “not delivered preterm 11.6%, indicating that almost 12% of SGA cases could have been prevented if mothers had not delivered preterm”, it would be good to add, in the discussion part, something about possible unmeasured confounding.

Response: We have added possible unmeasured confounding in the discussion (page 35, lines 459-462).

- The numbers in table 1, “pregnancy mode” look strange. The percentages do not add up to 100%.

Response: We agree with the reviewer, there was an error in the table, the table has been fully reviewed, and the error has been fixed (Table 1).

- In the conculsion you write: "SGA and LGA births are related multi-factor interactions of demographic and medical confounders that can be mediated by prematurity and gender of the baby". This sentence is un-clear. What do you mean? What do you mean with interactions?

Response: We do agree with the reviewer that the sentence is unclear. The indicated sentence has been rephrased (pages 36; lines 475-485).
